# Predictive Value of Neutrophil‐to‐Lymphocyte Ratio for Immune Checkpoint Inhibitor‐Related Myocarditis Among Patients Treated for Non‐Small‐Cell Lung Cancer

**DOI:** 10.1002/cai2.163

**Published:** 2025-02-20

**Authors:** Jian Xue, Chuanbin Liu, Jun Shao, Li Wang, Yating Han, Jing Wang, Jinda Wang

**Affiliations:** ^1^ Senior Department of Cardiology The Sixth Medical Center of Chinese PLA General Hospital Beijing China; ^2^ Western Medical Branch of Chinese PLA General Hospital Beijing China; ^3^ Department of Emergency The First Medical Center of Chinese PLA General Hospital Beijing China; ^4^ Department of General Surgery The First Medical Center of Chinese PLA General Hospital Beijing China

**Keywords:** biomarkers, immune checkpoint inhibitors‐related myocarditis, immunotherapy, neutrophil‐to‐lymphocyte ratio, non‐small‐cell lung cancer

## Abstract

**Background:**

The predictive value of the neutrophil‐to‐lymphocyte ratio (NLR) for immune checkpoint inhibitors (ICIs) in various tumors remains uncertain despite its use in forecasting the effectiveness of immunotherapy. The purpose of our research was to determine the prognostic significance of NLR for immune checkpoint inhibitor‐related myocarditis in non‐small‐cell lung cancer (NSCLC) patients.

**Methods:**

We enrolled and monitored patients with NSCLC who received ICI therapy at the Fifth Medical Center of Chinese PLA General Hospital between January 1, 2018, and February 20, 2021. NLR was determined before and soon after each cycle of ICIs. All participants in this study were periodically examined for troponin and brain natriuretic peptide (BNP), and an electrocardiogram (ECG) and echocardiography were done. Cox's proportional hazards regression model and receiver operating characteristic (ROC) were used to assess the predictive value for ICI‐related myocarditis.

**Results:**

A total of 146 patients received ICI treatment and completed a follow‐up. Of these, 17 patients (11.64%) developed ICI‐related myocarditis that met the diagnostic criteria. The initial cycle revealed that the NLR was a reliable predictor of potential myocarditis related to ICIs, with an area under the curve (AUC) of 0.833 and a 95% confidence interval (CI) of 0.721–0.945. Following the initial round of ICI treatment, an NLR elevation (NLR ≥ 3.25) appeared to be the most significant standalone indicator of ICI‐related myocarditis (HR: 11.094; 95% CI: 3.186–38.631; *p* < 0.001).

**Conclusions:**

Our study confirmed that NLR elevation in the early phase after ICI treatment of NSCLC is a reliable predictive factor of ICI‐related myocarditis. Regular and frequent cardiac monitoring may help to avoid the occurrence of severe and fatal cases.

## Graphical abstract

Our study found that the neutrophil‐to‐lymphocyte ratio elevation in the early phase after immune checkpoint inhibitors (ICIs) treatment in non‐small‐cell lung cancer is a predictive factor for ICI‐related myocarditis. Regular and frequent cardiac monitoring may help to avoid the occurrence of severe and fatal cases.

**Abbreviations**
AUC: area under the curve
BNP: brain natriuretic peptide
ECG: electrocardiogram
ICI: immune checkpoint inhibitor
LVEF: left ventricular ejection fraction
NLR: neutrophil‐to‐lymphocyte ratio
NSCLC: non‐small‐cell lung cancer
NT‐proBNP: N‐terminal pro‐B‐type natriuretic peptide
ROC: receiver operating characteristic

## 1 Introduction

Immune checkpoint inhibitors (ICIs) are a new type of medication that assists in guiding the immune system to identify and attack cancer cells, leading to significant improvements in lung cancer treatment results [1]. ICIs consist of drugs that target programmed death‐1 inhibitors, programmed death ligand‐1 inhibitors, cytotoxic T lymphocyte‐associated antigen 4, and so forth [1]. In recent years, the benefits of programmed death‐1 inhibitors have been expanding and it has become the first‐line treatment option for non‐small‐cell lung cancer (NSCLC) [2]. Although ICIs boost the body's immune reaction against tumors, they can also cause immune‐related adverse events affecting various organs, with cardiac immune‐related adverse events being the most fatal [3]. The prevalence of cardiac immune‐related adverse events was 1.3%, and myocarditis had the highest prevalence, accounting for 50.8% of all cardiac immune‐related adverse events [4]. Studies have shown that the incidence of ICI‐related myocarditis varies between 0.27% and 1.14%, with a potential mortality rate of up to 50% [5]. Additionally, ICI‐related myocarditis may be underestimated because of factors like nonspecific symptoms, the lack of specific markers, potential confusion with other heart conditions, and other challenges in diagnosis [6, 7]. With the wider use of ICIs, a corresponding increase in the number of patients with ICI‐related myocarditis is expected; however, effective clinical interventions are still lacking.

It has been demonstrated that immunotherapy for lung cancer raises the risk of cardiac events more than in other types of solid tumors. Within a year, lung cancer patients faced a 9.7% chance of experiencing cardiac events, such as myocarditis [8]. The mechanism by which ICI‐related myocarditis occurs in lung cancer patients is not fully understood. When damage to the heart occurs, cardiac autoantigens are exposed to T lymphocytes. Cardiomyocytes can inhibit T‐cell activation and invasion by upregulating immune checkpoints, and ICIs disrupt this cardiac immune homeostasis [5]. When lung cancer patients receive radiotherapy and immunotherapy at the same time, the immune response is enhanced, but it may lead to exposure of the patient's cardiac autoantigens [9, 10]. Treatment with ICIs suppresses the immuno‐protective mechanisms of the heart and activates T cells, leading to a higher incidence of cardiotoxicity [11]. NLR can be used as a marker to predict cardiovascular events. Increased NLR in heart disease is strongly associated with the effect of inflammation and oxidative stress [12]. Mosca et al. confirmed that NLR can reflect tumor‐induced inflammatory states and is a potential prognostic indicator for tumor immunotherapy [13]. Elevated NLR reflects a relative increase in the organism's neutrophil count and/or a relative decrease in the lymphocyte count. The increase in neutrophils represents an increase in the inflammatory response of the organism, which promotes a massive release of inflammatory mediators; however, neutrophils can also inhibit the activity of lymphocytes and natural killer cells, reduce the anti‐tumor immune response of the organism, and promote distant metastasis of the tumor. The reduced number of lymphocytes weakens the body's anti‐tumor immune function and decreases the effective immune response to tumors, leading to tumor invasion and development. In conclusion, elevated NLR suggests an imbalance in the body's anti‐tumor immune‐inflammatory response, which is a sign of poor prognosis [14]. NLR, when used in conjunction with biomarkers like programmed death ligand‐1 inhibitor, as well as tumor mutation burden, and lactate dehydrogenase, can serve as a predictive indicator of immunotherapy outcome in NSCLC [15, 16, 17]. Drobni et al. confirmed that individuals with ICI‐related myocarditis exhibit a higher NLR and are at greater risk of major adverse cardiac events compared with those without any immune‐related adverse events [18].

As the NLR value for predicting ICI‐related myocarditis in patients with NSCLC is unclear, we designed a prospective cohort study. In this study, we assessed the predictive value of NLR throughout treatment in patients with NSCLC who developed ICI‐related myocarditis.

## 2 Methods

### 2.1 Ethical Approval

This research followed the guidelines of the 1975 Declaration of Helsinki and was authorized by the Ethics Committee of The Fifth Medical Center of Chinese PLA General Hospital (2020‐5006‐03). Informed written consent was obtained from all participants.

### 2.2 Patients and Study Design

This research was a single‐center prospective observational study focusing on NSCLC conducted at The Fifth Medical Center of Chinese PLA General Hospital from January 1, 2018, to February 20, 2021. Individuals aged 18 years and above newly diagnosed with NSCLC were considered eligible for enrollment. Participants were excluded if they had a history of autoimmune diseases (with a few exceptions such as type 1 diabetes and well‐controlled thyroid disorders), heart failure, severe heart issues, interstitial lung disease or pneumonitis, severe infections, cancer diagnosis within the last 5 years, or were taking systemic immunosuppressive drugs [19]. Patients received programmed death‐1 inhibitors or programmed death ligand‐1 inhibitors according to the current guidelines. Patients were observed until the onset of the first myocarditis diagnosis or the end of the follow‐up period (12 months), whichever came first.

### 2.3 Diagnosis of ICI‐Related Myocarditis

In this study, we outlined the key criteria for diagnosing ICI‐related myocarditis on the basis of the Chinese Society of Cardiology guidelines for the diagnosis and treatment of adult fulminant myocarditis in 2024 [20] and the ESC Guidelines for cardio‐oncology [21] in 2022. These criteria included (1) clinical manifestations after ICI treatment, such as palpitation, chest pain, chest tightness, pericarditis, and pericardial effusion, were consistent with the characteristics of myocarditis; (2) ECG showed new sinus tachycardia, frequent ventricular premature beats, atrioventricular block or QT interval extension, ST‐segment elevation or T wave inversion, R wave amplitude reduction, or other abnormal manifestations after ICI treatment; (3) the level of brain natriuretic peptide (BNP) or troponin increased after ICI treatment; (4) decrease in left ventricular ejection fraction (LVEF) on an echocardiogram, or with changes such as abnormal wall motion, diffuse left ventricular systolic dysfunction, and cardiac enlargement after ICI treatment. If two or more criteria were met, ICI‐related myocarditis was evaluated. The decision to terminate or continue ICI treatment was at the clinical oncologists and cardiologists’ discretion.

### 2.4 Data Collection and Follow‐Up

Data on demographic characteristics, smoking history, coronary artery disease, hypertension, diabetes mellitus, lung cancer type, ICI regimens, targeted therapy combinations, and treatment‐related signs and symptoms were gathered. Upon admission, various laboratory tests were conducted, such as a full blood count, coagulation profile, serum biochemical panel, erythrocyte sedimentation rate, troponin, and NT‐proBNP.

Following admission, laboratory tests and imaging studies were performed repeatedly, with observations of signs and symptoms, treatments administered, and outcomes documented. In the first four cycles of ICI treatment, we reexamined the blood count. NLR was measured and calculated before and soon after each cycle of ICIs. All participants in this study were assessed for LVEF by a 2D echocardiography scan every month after treatment initiation. Additionally, every month, troponin and BNP were reexamined and an ECG was performed.

### 2.5 Statistical Analysis

For statistical evaluation, SPSS version 26.0 was used. Continuous variables with a normal distribution are expressed as mean ± SD, and No. (%) were used for categorical variables. Shapiro–Wilk normality test was used to assess the normality of the data distribution. For comparisons between two groups, unpaired 2‐tailed Student *t*‐tests or Mann–Whitney *U* tests were applied for normally or nonnormally distributed data, respectively. Categorical variables were compared by testing proportions with either the *χ* ^2^ test or Fisher's exact test. The predictive value of NLR was calculated using receiver operating characteristic (ROC) curve analysis. Stratification for predictors of ICI‐related myocarditis was performed using the Kaplan–Meier method, with comparisons between the myocarditis and normal groups performed using the log‐rank test. Univariable and multivariable Cox regression models were used to identify the risk factors linked to myocarditis related to ICIs. *p* < 0.05 was considered statistically significant.

## 3 Results

### 3.1 Characteristics of Patients

From January 1, 2018, to February 20, 2021, a total of 162 patients with NSCLC were assessed for potential participation in this research. There were eight individuals with heart failure, six with pneumonia, and two with systemic lupus erythematosus, resulting in a total of 16 patients being removed from the study group. Consequently, a total of 146 patients were included in the analysis of outcomes. Among all enrolled patients, the average follow‐up period was 10.73 months, and the longest follow‐up period was 12 months. The average age was 57.59 ± 14.78 years in the ICI‐related myocarditis group. Adenocarcinoma was the most common primary cancer type, accounting for 54.11% of cases, and only 22.22% of patients were receiving a combination of ICIs and targeted therapy. The study found that 17 patients (11.64%) developed myocarditis related to ICIs, meeting the diagnostic criteria. The prevalence of smoking, diabetes, and hypertension did not significantly differ between the two groups; however, a higher proportion of patients in the group with ICI‐related myocarditis (35.3% *vs.* 10.1%) had a history of coronary artery disease compared with the control group. The majority (52.9%) of individuals with ICI‐related myocarditis did not show any symptoms. Weakness and tachycardia, which lack specificity, were the most frequent symptoms observed upon admission. The incidence of ICI‐related myocarditis was higher in the group receiving ICIs combined with targeted therapy compared with the control group (35.3% *vs.* 20.2%), though the difference was not statistically significant (*p* = 0.156). Additionally, there was no significant difference in the primary cancer type and the ICI regimens between the two groups. Table 1 contains the characteristics of the patients with and without ICI‐related myocarditis.

**Table 1:** Characteristics of patients with and without ICI‐related myocarditis.

	Myocarditis (*n* = 17)	Controls (*n* = 129)	
Characteristic	No. (%)	No. (%)	*p*
Age at start of ICI (years)	57.59 ± 14.78	58.57 ± 13.74	0.768
Female	5 (29.41)	45 (34.88)	0.655
Cardiovascular risk factors			
Current or prior smoking	5 (29.41)	43 (33.33)	0.746
Hypertension	4 (23.53)	43 (33.33)	0.416
Diabetes mellitus	5 (29.41)	31 (24.03)	0.629
Coronary artery disease	6 (35.29)	13 (10.08)	0.004
Primary cancer type			0.974
Adenocarcinoma	9 (52.94)	70 (54.26)	—
Squamous cell carcinoma	5 (29.41)	39 (30.23)	—
Others	3 (17.65)	20 (15.50)	—
Signs and symptoms during treatment			
Symptomatic	9 (52.94)	22 (17.05)	0.001
Chest pain	1 (5.88)	5 (3.88)	0.695
Dyspnea	2 (11.76)	6 (4.65)	0.226
Tachycardia	5 (29.41)	15 (11.63)	0.045
Weakness	6 (35.29)	14 (10.85)	0.006
Others	1 (5.88)	7 (5.43)	0.938
ICI regimens			0.333
Cindilimab	5 (29.41)	32 (24.81)	—
Nivolumab	5 (29.41)	33 (25.58)	—
Pembrolizumab	3 (17.65)	25 (19.38)	—
Tislelizumab	2 (11.76)	17 (13.18)	—
Atezolizumab	1 (5.88)	15 (11.63)	—
Other	0 (0.00)	7 (5.43)	—
Combined targeted therapy	6 (35.29)	26 (20.16)	0.156
Pre‐ICI LVEF (%)	63.53 ± 11.45	65.22 ± 11.83	0.640
Pre‐ICI NT‐proBNP (ng/L)	70.24 ± 34.36	64.57 ± 21.34	0.344
Pre‐ICI NLR	2.55 ± 0.85	2.37 ± 0.89	0.437
NLR after 1st cycle	3.81 ± 0.77	2.79 ± 0.69	< 0.001
NLR after 2nd cycle	3.64 ± 0.76	2.85 ± 0.80	< 0.001
NLR after 3rd cycle	3.16 ± 0.87	2.94 ± 0.81	0.296
NLR after 4th cycle	2.92 ± 0.63	2.85 ± 0.85	0.749

Abbreviations: ICI, immune checkpoint inhibitor; LVEF, left ventricular ejection fraction; NLR, neutrophil‐to‐lymphocyte ratio; NT‐proBNP, N‐terminal pro‐B‐type natriuretic peptide.

### 3.2 Association Between NLR and ICI‐Related Myocarditis

After ICI therapy, the NLR of patients showed an ascending trend, but the increase in the ICI‐related myocarditis group was more apparent. Compared with the control group, the NLR was notably elevated in the group with ICI‐related myocarditis during the first and second cycles. During the third and fourth rounds of ICI therapy, the NLR level decreased, with no significant statistical variance observed between the two groups (Table 1).

**Figure 1:**
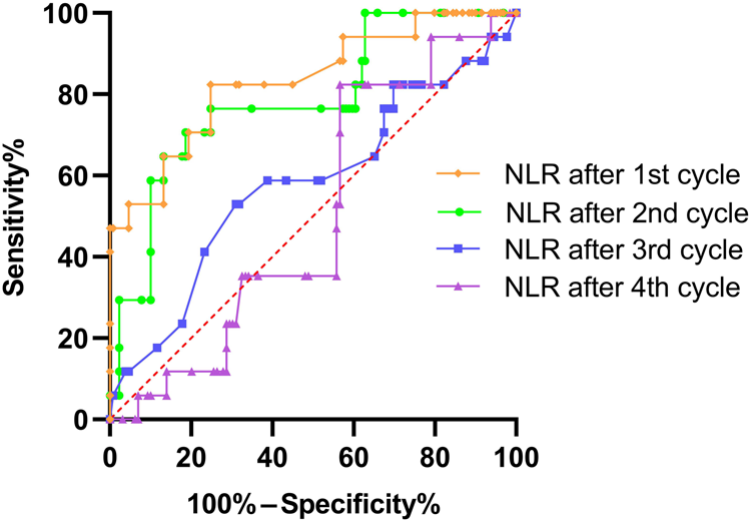
ROC curve analysis of the NLR after each cycle of different ICI treatments for predicting ICI‐related myocarditis. ICI, immune checkpoint inhibitor; NLR, neutrophil‐to‐lymphocyte ratio; ROC, receiver operating characteristic.

The diagnostic validity of the NLR in the different cycles was evaluated using the ROC curve (Figure 1). Based on ROC curve analysis, the NLR in the first cycle was a reliable predictor of ICI‐related myocarditis, with an AUC of 0.833 and 95% CI of 0.721–0.945. The AUC of the NLR in the second cycle was 0.786 (95% CI: 0.668–0.904). Moreover, we found that the optimal NLR cut‐off point was 3.25, considering as maximum values a sensitivity of 82.4% and a specificity of 75.2% (Youden index = 0.576).

**Figure 2:**
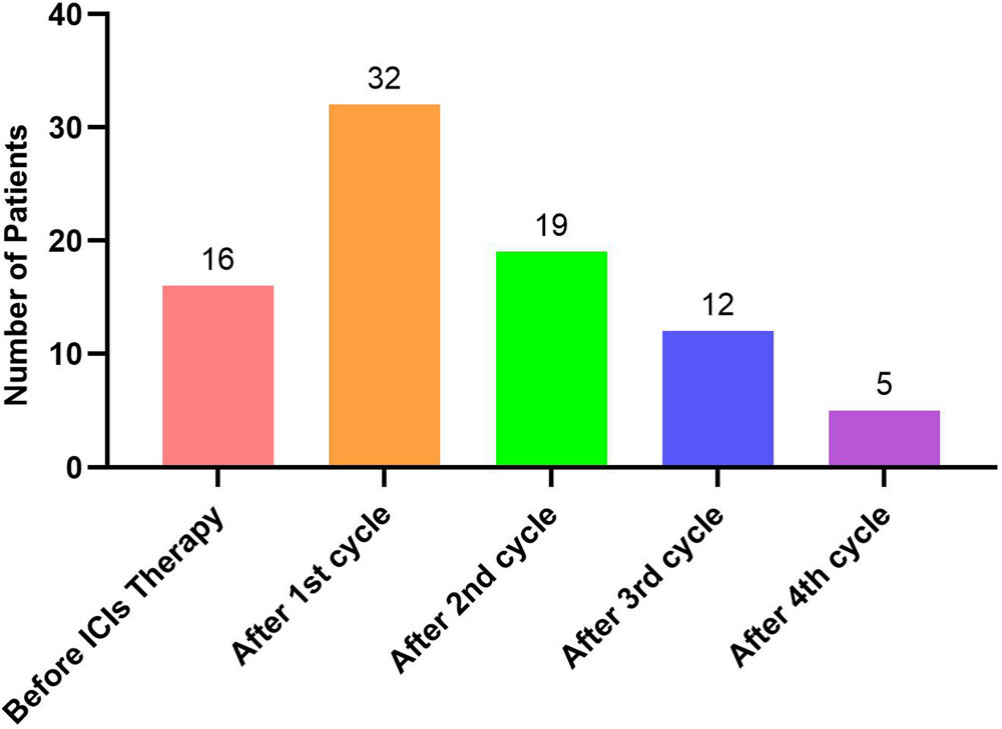
Time of the first detection of an elevated NLR value. ICI, immune checkpoint inhibitor; NLR, neutrophil‐to‐lymphocyte ratio.

Patients were categorized into two groups on the basis of the ROC curve analysis, with the cut‐off value for NLR set at 3.25 (normal group < 3.25; elevation group ≥ 3.25). NLR elevation (NLR ≥ 3.25) was found in 84 patients. NLR elevation was observed at baseline before the initiation of ICI treatment in 16 cases, which was potentially attributed to individual variances. Among the 68 patients left, the NLR was initially within the normal range but rose as they underwent ICI treatment. In the majority of instances, the initial increase in NLR was detected shortly after the first cycle of ICIs (Figure 2). Most patients experienced a temporary increase in NLR that returned to normal within four cycles. ICI‐related myocarditis was seen in the elevated NLR group between 1 and 8 months after the initial detection of NLR elevation.

**Figure 3:**
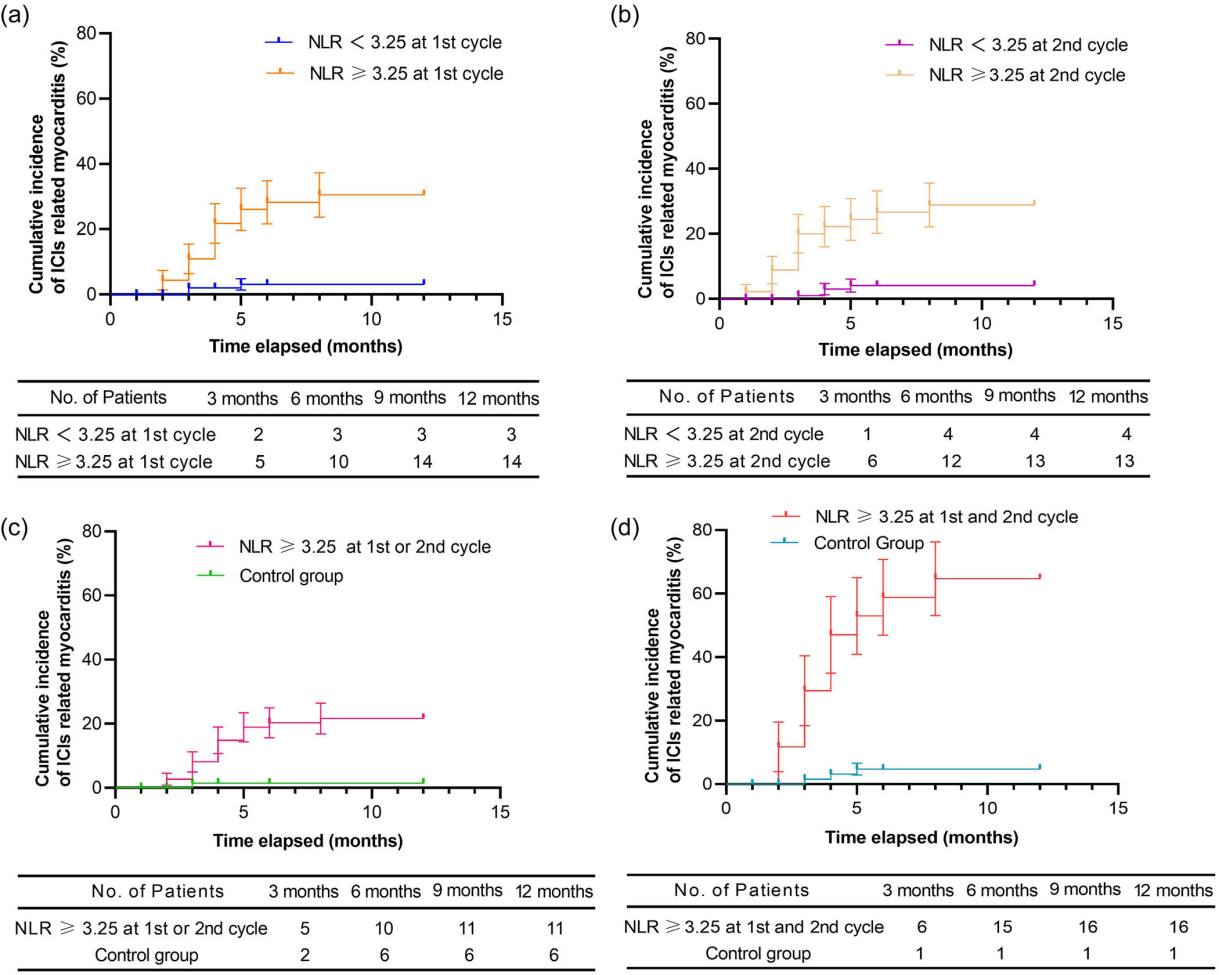
Kaplan–Meier curves of risk group stratification for the occurrence of ICI‐related myocarditis. (a) NLR elevation in the first cycle, (b) NLR elevation in the second cycle, (c) NLR elevation in the first or second cycle, and (d) NLR elevation in the first and second cycle. ICI, immune checkpoint inhibitor; NLR, neutrophil‐to‐lymphocyte ratio.

The Cox model for analyzing survival rate was used to assess the effect of cardiovascular risk factors and pathological NLR elevation on the association with ICI‐related myocarditis (Table 2). Based on the univariable analysis, the symptoms of weakness (HR: 4.120; 95% CI: 1.518–11.179; *p* = 0.005) and tachycardia (HR: 3.013; 95% CI: 1.060–8.568; *p* = 0.039) during ICI therapy may be important predictors of ICI‐related myocarditis. Additionally, multivariate analysis showed that the elevation of NLR in the first and second cycles has an important predictive value for the occurrence of subsequent ICI‐related myocarditis. After the first round of ICI treatment, the rise in NLR was the most significant factor in predicting ICI‐related myocarditis (HR: 11.094; 95% CI: 3.186–38.631; *p* < 0.001). Finally, patients were categorized into four groups on the basis of their NLR during the initial and subsequent cycles of ICI treatment: NLR elevation in the 1st and 2nd cycle; NLR elevation in the 1st or 2nd cycle; NLR elevation in the 1st cycle; NLR elevation in the 2nd cycle. Figure 3 displays the progressive incidence of myocarditis related to ICIs over time, according to Kaplan–Meier estimates of the four groups. Interestingly, continuous NLR elevation (HR: 19.102; 95% CI: 7.018–51.995; *p* < 0.001) emerged as a powerful predictor of ICI‐related myocarditis, highlighting the crucial clinical relevance of repeated NLR assessments in patients undergoing ICI therapy.

**Table 2:** Cox regression mode using risk factors associated with ICI‐related myocarditis.

Characteristic	Univariable HR (95% CI)	*p*	Multivariable HR (95% CI)	*p*
Age (years)	—	0.279	—	—
< 65	Ref	—	—	—
≥ 65	0.539 (0.176–1.652)	—	—	—
Female	0.798 (0.281–2.266)	0.672	—	—
Current or prior smoking	0.808 (0.285–2.294)	0.689	—	—
Hypertension	0.604 (0.197–1.854)	0.379	—	—
Diabetes mellitus	1.256 (0.443–3.565)	0.668	—	—
Coronary artery disease	2.109 (0.954–4.660)	0.065	1.237 (0.338–4.526)	0.748
Primary cancer type		0.845	—	—
Adenocarcinoma	1 (Ref.)	—	—	—
Squamous cell carcinoma	0.878 (0.238–3.243)	—	—	—
Others	0.828 (0.198–3.466)	—	—	—
Signs and symptoms at disease onset		—	—	—
Symptomatic	4.846 (1.867–12.573)	0.001	3.169 (0.617–16.266)	0.167
Chest pain	2.060 (0.273–15.571)	0.484	—	—
Dyspnea	2.598 (0.593–11.377)	0.205	—	—
Tachycardia	3.013 (1.060–8.568)	0.039	1.823 (0.334–9.955)	0.488
Weakness	4.120 (1.518–11.179)	0.005	0.425 (0.083–2.168)	0.304
Others	1.361 (0.180–10.263)	0.765	—	—
Combined targeted therapy	2.084 (0.770–5.638)	0.148	2.018 (0.496–8.215)	0.327
Pre‐ICI NLR	1.248 (0.407–3.826)	0.699	—	—
NLR after 1st cycle	11.094 (3.186–38.631)	0.000	12.078 (2.933–49.742)	0.001
NLR after 2nd cycle	7.967 (2.597–24.445)	0.000	10.842 (2.991–39.301)	< 0.001
NLR after 3rd cycle	1.902 (0.724–4.997)	0.192	1.246 (0.344–4.508)	0.738
NLR after 4th cycle	0.721 (0.267–1.949)	0.519	0.765 (0.226–2.597)	0.668

Abbreviations: ICI, immune checkpoint inhibitor; NLR, neutrophil‐to‐lymphocyte ratio.

## 4 Discussion

In this study, we prospectively evaluated the rate of ICI‐related myocarditis among patients with NSCLC in a single center and explored the association of NLR in the different cycles of ICI therapy with myocarditis. We found that the NLR elevation in the early phase after ICI treatment in NSCLC is a reliable predictive factor of ICI‐related myocarditis.

Our research found that the incidence of ICI‐related myocarditis in lung cancer patients was 11.64%, which is higher than the previously reported range of 0.06%–1.14% [22, 23, 24]. Nevertheless, over the last 3 years, there has been a rise in the number of myocarditis cases linked to the use of ICIs. In a World Health Organization's global population‐based retrospective study of 31,321 cancer patients treated with ICIs, 5,515 (17.61%) cases were reported to have ICI‐related myocarditis [25]. Furthermore, prior studies have primarily relied on historical data from a single center, making it difficult to implement thorough systematic monitoring and timely identification of ICI‐related myocarditis. Regular surveillance for myocarditis is not commonly conducted in individuals undergoing treatment with ICIs in clinical practices, potentially resulting in its underrepresentation. We used a prospective observation cohort study, which has three main advantages. First, compared with the retrospective cohort study, we controlled and screened the enrolled cases to reduce bias factors. Second, in addition to the systematic screening of the enrolled cases, the researchers conducted patient observation and follow‐up throughout the entire process of enrolling patients, effectively removing some possible bias factors. Third, there are many uncertainties in the retrospective study. Patients in this research were examined during each cycle throughout the treatment to confirm the accuracy of the observation endpoint. Furthermore, there is an ongoing debate regarding the diagnostic standards for myocarditis related to ICIs in multiple studies, leading to varying incidence rates of this condition across different research projects.

Our results indicated that the cases of ICI‐related myocarditis were mild and did not affect mortality. Only 8 (47.1%, 8/17) patients with ICI‐related myocarditis had symptoms such as weakness and tachycardia. There was no severe or lethal case in our study. Contrary to previous reports of a 30%–50% fatality rate associated with myocarditis related to ICI treatment [26], our research revealed a different outcome. One possible theory is that consistent and frequent cardiac examinations during monitoring could result in earlier identification of myocarditis related to ICIs [27]. Additionally, multiple studies have indicated that myocarditis is more common and severe when using combination immunotherapy, such as ipilimumab and nivolumab together [28, 29]. However, in our study, there was no combined immunotherapy, which may also be the reason for the absence of severe cases.

Serum troponin and electrocardiogram are currently proposed to detect myocarditis; however, these methods lack specificity [22]. It has been demonstrated that global longitudinal strain under echocardiography can be used to predict subclinical cardiotoxicity of treatment with ICIs [30, 31], but it may entail additional costs. In contrast, NLR can easily be derived during routine examination of complete blood counts and classification, making it accessible and inexpensive; therefore, it was chosen for our study. Our research showed that NLR can accurately predict myocarditis following ICI treatment, specifically the onset and severity of the condition as early as the first and second treatment cycles. Furthermore, our findings demonstrated that the increase in NLR following treatment with ICIs enables us to stratify the risk of myocarditis in cancer patients over the following year. Inflammatory cells play a crucial role in the progression of immune‐related adverse events, and therefore systemic inflammation markers can help predict the outcome [32]. The exact mechanism responsible for the increase in NLR after treatment with ICIs is not known, but it is speculated that it may be related to myocardial injury. Because of the distinct nature of the mechanism behind myocarditis compared with cardiotoxicity from traditional chemotherapy, elevation of this marker complicates the beginning of excessive inflammatory response. Prior research has indicated that the signaling pathways associated with neutrophil activation and the creation of neutrophil extracellular traps can affect the onset and progression of myocarditis [33]. Furthermore, lymphocytes play a crucial role in guiding and enhancing the adaptive immune response, offering a wider and more precise range of recognition of both self and non‐self‐antigens [34]. Histologically, individuals experiencing myocarditis related to ICIs display acute lymphocytic infiltrations into the heart muscle, suggesting the involvement of T‐cell‐mediated processes [28]. New findings have revealed how NLR is linked to a negative outcome in individuals with cancer. A high NLR of ≥ 3.25 was a significant indicator of poor prognosis in patients who had undergone complete resection of centrally located lung squamous cell carcinoma [35, 36]. A comprehensive study involving a large population is necessary to explore the importance of NLR in the occurrence of myocarditis related to ICIs.

This is the first study to suggest that the NLR in peripheral blood is a potential predictor of ICI‐related myocarditis in NSCLC patients. Peripheral blood is a valuable source of immunological data. Alterations in the composition of immune cells, such as neutrophils and lymphocytes, which can be easily identified in full blood counts, may indicate the equilibrium between the body's immune response and the presence of pathogens to some degree. This study also has several limitations, such as the small number of patients enrolled, which reduces statistical efficacy. Another limitation is that the diagnostic criteria for ICI‐related myocarditis were not clear. Diagnosing myocarditis related to ICIs on a large scale poses a challenge owing to the complexity and potential dangers associated with acquiring heart biopsy samples for precise diagnosis [37]. The evaluation of cardiac damage assessed by 2D echocardiography and serum cardiac markers (e.g., cardiac troponin T/I and NT‐proBNP) was deemed inadequate for monitoring cardiac function in this study. Therefore, a more comprehensive imaging technique is necessary in future studies.

## 5 Conclusions

The incidence of ICI‐related myocarditis in clinical practices was found to be significantly higher than previously reported results and therefore may be greatly underestimated. Regular and frequent cardiac monitoring may help to reduce serious and fatal cases. The NLR elevation in the early phase after ICI treatment of NSCLC is a reliable predictive factor of ICI‐related myocarditis.

## Author Contributions

**Jian Xue:** investigation (equal), writing – original draft (equal). **Chuanbin Liu:** data curation (equal), writing – original draft (equal). **Jun Shao:** data curation (equal), investigation (equal). **Li Wang:** data curation (equal), formal analysis (equal). **Yating Han:** data curation (equal). **Jing Wang:** formal analysis (equal), writing – original draft (equal). **Jinda Wang:** formal analysis (equal), writing – original draft (equal).

## Ethics Statement

The research followed the guidelines of the 1975 Declaration of Helsinki and was authorized by the Ethics Committee of The Fifth Medical Center of Chinese PLA General Hospital (2020‐5006‐03).

## Consent

Informed written consent was obtained from all participants.

## Conflict of Interest

The authors declare no conflicts of interest.

## Data Availability

The data that support the findings of this study are available from the corresponding author upon reasonable request.
